# Circulating Tumor Cells for Glioma

**DOI:** 10.3389/fonc.2021.607150

**Published:** 2021-03-10

**Authors:** Huikai Zhang, Fanen Yuan, Yangzhi Qi, Baohui Liu, Qianxue Chen

**Affiliations:** Department of Neurosurgery, Renmin Hospital of Wuhan University, Wuhan, China

**Keywords:** glioma, circulating tumor cells, liquid biopsy, blood, biomarker

## Abstract

Liquid biopsy has entered clinical applications for several cancers, including metastatic breast, prostate, and colorectal cancer for CTC enumeration and NSCLC for EGFR mutations in ctDNA, and has improved the individualized treatment of many cancers, but relatively little progress has been made in validating circulating biomarkers for brain malignancies. So far, data on circulating tumor cells about glioma are limited, the application of circulating tumor cells as biomarker for glioma patients has only just begun. This article reviews the research status and application prospects of circulating tumor cells in gliomas. Several detection methods and research results of circulating tumor cells about clinical research in gliomas are briefly discussed. The wide application prospect of circulating tumor cells in glioma deserves further exploration, and the research on more sensitive and convenient detection methods is necessary.

## Introduction

Glioma is the most common malignant brain tumor, with 200,000 patients diagnosed worldwide each year (1). Among all primary central nervous system tumors, 47.7% of those are glioblastomas (GBM), its incidence increases with age, and its characteristic histopathology include high mitotic activity, extensive necrosis and microvascular hyperplasia (2–4). Glioma has a poor prognosis and often relapses. Over time, the degree of malignancy tends to increase. Most brain malignant tumors are mainly treated with surgical resection, combined with radiotherapy, chemotherapy, and other comprehensive treatment methods, but the current treatment effect is poor. The median overall survival of high-grade glioma is 14–17 months (1). At present, the main auxiliary diagnostic means are imaging evaluation and pathological diagnosis. The treatment effect of glioma patients is evaluated by the response to tumor radiological appearance on MRI images (5, 6). However, there is a response of treatment-related brain tissue to chemotherapy and radiation therapy, known as pseudoprogression, and the pseudoprogression can cause enhancements and edema increase just like tumor progression on MRI (7, 8). The condition may be caused by treatment-related localized inflammation, which leads to edema and increased abnormal vascular permeability. At present, there are no biomarkers or imaging or clinical patterns that can reliably distinguish glioma recurrence from pseudoprogression, or monitor treatment effectiveness.

Recently, there has been a new minimally invasive tool to get information about tumor: liquid biopsies (9). Different types of circulating biomarkers that were associated with tumor, were identified in the serum and cerebrospinal fluid, including circulating tumor cells (CTCs), exosomes (microvesicles), circulating nucleic acids (DNA, RNA, and miRNA), proteins, and metabolites (10). These biomarkers, which rely on reliable detection means in the blood or cerebrospinal fluid, are relatively easy to obtain, allow for repeated measurements, and are significantly better to monitor disease.

Currently, many techniques have been developed for separating CTC from abundant leukocytes and erythrocytes. The physical-based separation depends on the size, deformability, density, or the dielectric properties of CTC different from normal blood cells (11). Epithelial cell adhesion molecule (EpCAM) is often used as a target for CTC enrichment because it is widely expressed on the cell surface of cancer cell-derived CTCs and has not been detected on normal blood cells (12). Anti- EpCAM antibodies can be used to directly detect or enrich CTC, while removing other cells by using anti-CD45 antibodies (specific for leukocytes). Different types of nanomaterials, such as magnetic nanoparticles (MNPs), gold nanoparticles (AuNPs), which can promote cell adhesion and have been reported for CTC detection (13). A variety of detection methods are used for CTC detection, but due to the complexity of the operation or lack of specificity, CTC cannot be widely used. Fast, convenient, and highly specific detection methods are still the limitations of CTC research.

Liquid biopsy has entered clinical applications for several cancer types, including breast and colorectal cancer, and has improved the individualized treatment of many types of cancer, but relatively little progress has been made in validating circulating biomarkers for brain malignancies. This review outlines the current status of the developments of circulating tumor cells for glioma.

## Circulating Tumor Cells

Circulating Tumor Cells (CTCs) are tumor cells which existing in the human circulatory system and have three typical characteristics: Originating from the primary tumor or metastases; Appearing in blood vessels and Participating in blood circulation (14). They are considered to be extremely harmful because it is an important cause of distant metastasis and death of malignant tumors. CTCs exhibit a variety of advanced properties, such as epithelial-mesenchymal transition (EMT) and dormancy, that are essential to support their survival in the bloodstream, radiological, and chemical resistance, escape from the anti-cancer immune response, and metastasis (15). CTCs that from primary or metastatic lesions and with long-term survival in the blood circulation system represent the most aggressive subcloning of tumor cells. Precise molecular typing of these tumor cells can help clinicians to diagnose metastatic or recurrence, and can be used to monitor the degree of disease progression and treatment response. However, most CTCs are rapidly destroyed in the circulation, and the metastatic potential appears to be limited. Only 2.5% of CTCs were shown to form micrometastases, and only 0.01% of these cells can induce macrometastases (16).

A large number of clinical trial data have proved that CTC is an independent prognostic factor, a marker and target for targeted therapy in patients with advanced breast cancer, lung cancer and prostate cancer (17–19). Though peripheral metastasis to glioma is considered rare, CTCs have been detected in the blood of glioma patients (20), and many isolated cases of glioma metastasis have been reported (21). Therefore, the identification of CTCs in glioma patients has important clinical application value for early tumor diagnosis and the prognosis. Even many molecular characteristics of gliomas such as the combined deletion of 1p/19q, IDH1 mutations, and hypermethylation of the MGMT promoter are expected to be detected in circulating tumor cells in peripheral serum of glioma patients. So far, data on CTCs about glioma are limited, the application of CTCs as biomarker for glioma patients has only just begun, and the requirements for the specificity of the detection technology are more important.

## Detection Methods of CTCS in Glioma

### GFAP-Positive as a Marker

Muller et al. used immunostaining against GFAP as a GBM marker and detected CTCs in 29 of 141 (20.6%) GBM patients (22). Glial fibrillary acidic protein (GFAP) is the main intermediate filament of mature and developing astrocytes, and is the most specific diagnostic marker for GBM cells. The serum concentration of GFAP measured by enzyme-linked immunosorbent assay is considered as a diagnostic marker for GBM (23, 24). The GFAP-positive cells be used for gene sequencing analysis and then found that the detected CTC cells had EGFR gene amplification similar to that of the primary tumor, deletion of chromosome 7 and 10 fragments, etc.

However, the expression of GFAP is not limited to glial cells, such as hepatic stellate cells (25), so it is necessary to eliminate interference from a small fraction of GFAP^+^ cells from other cells. At the same time, the study is limited to GBM, and the positive rate is very low.

### Telomerase-Based Assay

Kojima and colleagues engineered an adenovirus containing the gene for GFP that can selectively replicate in cells that express telomerase (26). MacArthur et al. (27) used a telomerase-responsive adenoviral probe that consist of the expression cassette for GFP and hTERT promoter, to detect CTCs. Telomerase has a catalytic subunit with reverse transcriptase activity (hTERT), and its expression is almost exclusively confined to cancer cells (28, 29). Telomerase and hTERT expression are closely related in human cancers but not observed in most normal tissues (30). Because well-differentiated WBCs typically do not express telomerase, the fluorescence detected can help distinguish CTCs from non-cancerous cells found in the blood. At the same time, experimentally verified that the tumor cells with increased telomerase activity detected by the probe are consistent with the expression of Nestin and EGFR, which are overexpressed in gliomas (31–36). The major advantage of the telomere-based assay is that this method has high sensitivity (more than 90% of solid tumors, including gliomas, increased expression of telomerase) and high specificity (telomerase not expressed in highly differentiated and normal cells) (30), but the operation is complicated and expensive. Large sample validation is still required.

### Examination for Aneuploidy of Chromosome 8 by FISH

Chromosome aberrations are unique characteristics of tumors and aneuploidy changes may lead to tumor development (37–44). The degree of chromosomal aberrations usually increases with tumor progression and is associated with poor prognosis (45). Tumors with aggressive clinical behavior are more likely to be aneuploid. Many studies have found that chromosome 8 has aneuploidy in many solid tumors, such as lung cancer, pancreas cancer, gastric cancer, colon cancer, and bladder transitional cell carcinoma et al., which provides feasibility for the detection of aneuploidy-based CTCs (46–50).

Fluorescence *in situ* hybridization (FISH) analysis is a powerful tool for detecting numerical and structural chromosome aberrations using chromosome specific DNA probes on interphase nuclei of various tumors. Gao et al. used the CEP8-FISH to detect CTCs in the peripheral blood of glioma patients (20). And CTCs were detected in different pathological subtypes of glioma, which indicated CTCs should be the common property of glioma. The detection method does not depend on surface-related antigen expression or cell size of glioma cells, and has good sensitivity.

### CTC-iChip and Antibodies Cocktail

The CTC-iChip is an integrated microfluidic platform, with its ability to isolate CTCs in suspension using both tumor antigen–dependent and tumor antigen–independent modes, is compatible with high-definition imaging and single-cell molecular analyses, as well as standard clinical cytopathology (51).

The CTC-iChip uses tagged antibodies against leukocyte surface markers CD45 and CD16 to efficiently remove leukocytes from blood, thereby enriching CTCs for immunofluorescence staining and molecular typing. Sullivan et al. developed a variety of specific antibodies that would identify GBM cells and complete absence in normal blood cells (52). Researchers chose five GBM specific antibodies including SOX2, Tubulin beta-3, EGFR, A2B5, and c-MET, annotated as STEAM, for simultaneous immunofluorescence staining. In the 33 GBM patients, only 39% had detected CTCs at least once in an average 2.6 venous blood draws.

### Recombinant VAR2CSA Malaria Protein

VAR2CSA malarial protein (rVAR2) has been used for CTC separation (53). The protein specifically binds to a unique chondroitin sulfate structure that is named oncofetal chondroitin sulfate (ofCS), which exists in the placenta and almost all cancer cells, and has limited expression in normal tissues (54). Bang-Christensen et al. (55) invented a CTC separation method that used magnetic beads to equip rVAR2 protein to capture CTCs from glioma patient blood samples. The study used ofCS as a target, not only for capture, but also for the detection of glioma CTC gene mutation. Only 10 glioma patient blood samples were tested. And there is no correlation between the number of CTCs and WHO grades.

### hTERT Promoter-Regulated Oncolytic Herpes Simplex Virus-1

Adusumilli et al. (56) reported the use of tumor-selective HSV expressing GFP to detect cancer cells in body fluids. HSV-1 can targets cancer cells with telomerase-reverse-transcriptase-positive and recognize CTCs by expressing green fluorescent protein. Zhang et al. (57) established a highly applicable method based on oHSV1-hTERT-GFP, to selectively label CTCs in peripheral blood by expressing GFP. It can be monitored by flow cytometry or fluorescence microscopy and more accurately determine the number of CTCs in the peripheral circulation of glioma patients, and may determine the molecular typing and genetic profiling.

### Other Methods

Krol et al. (58) used the Parsonrtix microfluidic cassette to ensure label-free physical capture of CTCs and CTCs were stained with a dedicated GBM CTC antibody cocktail containing antibodies against EGFR, Ki67, and EB1, as well as against the WBC marker CD45 to exclude leukocyte contamination. A novel aptamer combined with nanostructures improves affinity of CTCs capture (59). And a label-free separation microfluidic device was developed using multistage channel, which took full advantage of inertial lift force (60). These two methods are used to separate the mixture of human peripheral blood and glioma cell lines, which has high separation efficiency, simple operation and extremely low cost.

In fact, a wide variety of CTC enrichment approaches have been developed, but rarely applied to detect glioma cells. Due to the overlapping expression of markers in tumor cells and normal cells, the use of lineage markers to identify CTCs has some limitations. Further identification of CTCs at the DNA, RNA or protein level will help improve detection rates. There is still a need for more sensitive detection methods as well as clinical studies of large numbers of samples (Tables 1, 2 shows more details about detecting methods of CTCs in glioma patients).

**Table 1:** Circulating tumor cells in glioma patients.

**References**	**Source (ml)**	**Patients types (T/N)**	**Time of take**	**Cell enrichment**	**Cell characterization**	**Results**	**Treatment response**	***P*-value**	**Survival**
Muller et al. (22)	Blood 10 ml	GBM 141/23	Pre/postoperative	MNCs isolated by gradient centrifugation Erythrocyte lysis	Fluorescence immunocytochemistry GFAP+ and CD45-	*N* = 29/141 (>1CTCs/10 ml) **+**	Pre/postoperative (13.4%) Preoperative (6%) Postoperative (7.5%)	*P* > 0.05	Mean 13.1 Months *P* > 0.05
MacArthur et al. (27)	Blood 10 ml	WHO III/IV 20/30	Pre/post-radiotherapy	Centrifuge in OncoQuick tubes	telomerase-responsive adenoviral probe	*n* = 8/11 pre-radiotherapy *n* = 1/8 post-radiotherapy (>1.3CTCs/ml) **+**	CTCs (Pre-RT> Post-RT)	*P* < 0.001	NA
Gaoet al. (20)	Blood 7.5 ml	WHO II-IV 31/10	Pre/postoperative Pre/post- radiotherapy/ Chemotherapy	Centrifugation magnetic separation of beads	Immunostaining GEP8-FISH CD45–, GEP8+, GFAP–/+	*N* = 24/31 CTCs = 1–10/7.5 ml No statistically significant difference with grades	*n* = 1/9 After-surgery/radiotherapy/ Chemotherapy CTC = 1/7.5 ml	*P* < 0.01	NA
Sullivan et al. (52)	Blood 10–20ml	GBM 33/6	Pre/postoperative	CTC-iChip (magnetically tagged CD45/CD16)	Isolate single CTCs IHC glioma marker panel (STEAM) STEAM+, CD45–	*n* = 13/33 CTCs = 11.8 (12 progressive) CTCs = 2.1(21 stable) (>7CTCs/ml) **+**	NA	*P* < 0.001	NA
Bang-Christensen et al. (55)	Blood 3 ml	WHO II-IV 10/NA	NA	Erythrocyte lysis Centrifugation Incubated with rVAR2-coated magnetic beads	Immunostaining DAPI+, CD45/CD66b–,rVAR2+	*n* = 10/10 CTCs = 0.5–42/3 ml No statistically significant difference with grades	NA	NA	NA
Zhang et al. (57)	Blood 4 ml	WHO II-IV 39/178	NA	Erythrocyte lysis Centrifugation Resuspended with oHSV1-hTERT-GFP and anti-CD45	Flow cytometry CD45–/GFP+	*n* = 23/39 CTCs = 6.5/4 ml (>3CTCs/4 ml) **+**	NA	NA	NA
Krol et al. (58)	Blood 10 ml	GBM 13/3	Drug (BAL101553) pre-dose (7d, 0 h) post-dose (2 h, 24 h, 8d,22d)	Parsortix microfluidic device	Immunostaining EGFR+, Ki67+, EB1+, CD45–	*n* = 7/10 (>3CTCs/10 ml) **+**	Pre = 12 CTCs Post = 14 CTCs	*P* = 0.462	NA

*T, tumor; N, Normal; RT, radiotherapy; GBM, glioblastoma; MNCs, monocytes; NA, Not mentioned*.

**Table 2:** Advantages and disadvantages of the detection of the CTCs.

**Techniques**	**Advantages**	**Disadvantages**
Kojima et al. (26)	Simple, convenient, Easy to operate GFAP-positive cells be used for gene sequencing analysis	Detection rate is low (20.6%, 29/141) GFAP expression is not totally restricted to glial cells, such as hepatic stellate cells Insufficient sample size, Detection is limited to GBM
MacArthur et al. (27)	High sensitivity Telomerase not expressed in highly differentiated and normal cells Epithelial cell marker independent	Operation is complicated and expensive Insufficient sample size, Detection is limited to GBM Unable to mark genetic phenotype
Gao et al. (20)	Good sensitivity and specificity Detection in multiple grades of glioma Not depend on physical and chemical characteristics Polyploidy rate reflects the degree of malignancy	Operation is complicated and expensive Insufficient sample size
Sullivan et al. (52)	High sensitivity Determine the molecular typing and genetic profiling	Detection rate is low (39.3%, 13/33) Insufficient sample size, Detection is limited to GBM Increases the risk of false positives and high background levels
Bang-Christensen et al. (55)	High detection rate (100%, 10/10) Determine the molecular typing and genetic profiling	Insufficient sample size Increases the risk of false positives
Zhang et al. (57)	High sensitivity Epithelial cell marker independent Determine the molecular typing and genetic profiling	Insufficient sample size Unstable detection
Krol et al. (58)	High separation efficiency, simple operation and extremely low cost High sensitivity	Insufficient sample size

## Characterization of CTCs in Glioma

### Stem Cell-Like Properties

Liu et al. (61) reported that GBM-derived CTC possessed a cancer stem cell (CSC)-like phenotype and contributed to local tumorigenesis and recurrence by the process of self-seeding. CSCs are characterized by strong self-renewal ability and higher malignant potential (62), and also show advanced resistance to radiation and chemotherapy (63). And CSCs can enter the circulation from the tumor body as CTC, survive in focal and systemic treatments, and reseed seeds for the primary tumor bed to generate new local tumors, providing an alternative source for tumor *in situ* invasion. At present, it is speculated that CSC-like CTCs may return to the tumor bed through this replay mechanism and refill, which may cause tumor recurrence or metastasis (61). Similarly, clinical and surgical pathological evidence suggests that GBM metastases are common in the brain, which may not be fully explained by direct invasion of tumor cells (64). We speculated that the continued spread of CTC on the primary tumor bed may lead to local micrometastasis. In addition, glioma CSCs have significantly up-regulated Wnt signaling, which is essential for maintaining cell survival, inducing EMT, and tumorigenicity (65). Therefore, the elimination of CTCs with Wnt as the target may GBM's new treatment strategy.

### Mesenchymal Enrichment

GBM has been classified by The Cancer Genome Atlas (TCGA) Consortium into four subtypes: classic, mesenchymal (MQ), proneural (PN), and neural (66). The classic GBM shows high proliferation characteristics, often accompanied by high-level EGFR amplification. MQ is defined by overexpression of markers of MQ (CHI3L1/YKL40 and MET) and astrocytes (CD44 and MERTK), plus deletion or mutation of NF1 (17q11.2). PN show simultaneous activation of oligodendrocytes (PDGFRA, OLIG2, TCF3, NKX2-2) and PN (SOX, DCX, DLL3, ASCL1, TCF4) development-related genes, and are characterized by molecular changes in TP53, PDGFRA, and PIK3CA/PIK3R1 and IDH1 mutations. Neural-GBM lack a unique genetic map showing gene expression characteristics similar to those in normal brain tissue, and expressed neuronal markers such as NEFL, GABRA1, SYT1, and SLC12A5 (67–70).

Sullivan et al. (52) found that CTCs detected in GBM patients overexpress interstitial transcription proteins, and showed the transcriptional characteristics of mesenchymal GBMs. Neural/mesenchymal RNA-ISH assay showed that primary subpopulations of GBM cells which expressing high interstitial expression profiles were more likely to invade the bloodstream. These observations increase the possibility that MQ-GBMs will more easily enter the vascular lumen of the brain and circulate through the systemic vascular system.

### Clinical Value of CTCs

At present, there are few studies on CTCs in gliomas, and its clinical value cannot be fully determined due to unclear detection methods and insufficient clinical sample sizes. But the current research still yields some promising results.

Gao et al. detected CTCs in all glioma pathological subtypes (astrocytoma; oligodendroglioma; oligoastrocytoma; anaplastic astrocytoma; anaplastic oligodendroglioma; anaplastic oligoastrocytoma; GBM), and the number of CTCs increased with increasing malignancy; Muller's study found that the number of CTCs in patients after chemotherapy or radiotherapy was significantly lower than before, CTCs detection results were always consistent with the MRI findings in both radionecrosis and progression. CTCs can be regarded as a complement of MRI and it, to some extent, is superior to MRI in monitoring the treatment response and local microenvironment of glioma; James et al. tested the expression characteristics of a single CTC, which provided the possibility to determine the molecular characteristics of gliomas, and it is possible to determine the patient's pathological type and develop a treatment plan without invasive operations (20, 22, 52).

Almost all studies found that the CTCs count before and after the operation were basically the same, which may indicate that the surgical operation did not lead to the release of CTCs. Due to the shortage of sample size and follow-up time, the relationship between CTCs and disease progression and survival has not been observed. The challenge in limiting the use of CTC detection for gliomas in clinical applications lies in the complexity and low sensitivity of the detection technology. But there is no doubt that CTC has a incredible application prospect in intracranial malignancies.

## Research Challenges of CTCs in Glioma

### Blood-Brain Barrier Escape Mechanism

In the past few decades, the application of CTCs has not received much attention in glioma. Malignant glioma is highly aggressive, but its extracranial metastasis is rare. Because of the blood-brain barrier, there is a misconception that glioma cells can never enter the blood circulation. And recent researches have overturned this conclusion. So far, the mechanism of CTCs escape from the blood-brain barrier has not been fully elucidated.

Studies have suggested that some characteristics of CTCs may be the reason for their aggressiveness. CTCs that expressing high interstitial characteristics are more likely to invade the bloodstream (52). Some characteristics of GBM also help CTCs to enter the bloodstream and spread further throughout the body. GBM has the highest degree of vascularization compared to other types of tumors, and studies have found that the integrity of the blood-brain barrier in GBM patients is damaged, there are multiple windows and damaged tight connections (71, 72). Current findings are limited to GBM. It is worth noting that there is still a lack of comprehensive and in-depth understanding of this mechanism, especially in gliomas with lower WHO classification.

### Rare Extracranial Metastases

Although glioma cells can invade the bloodstream, they rarely turn into metastatic lesions outside the brain. Several reasons for low incidence of extracranial metastases of glioma have been discussed, include: Short survival time of patients, especially GBM; the Blood-brain barrier; the immune system inhibits the extracranial growth of glioma cells (73); There is no lymphatic channel in the central nervous system (74), or glioma cells cannot invade the connective stroma (75). Research by Yu et al. found that tumor cells can express PTEN (an important tumor suppressor) in peripheral organs, but once transplanted into the brain, the expression of PETN decreases (76). And in the brain, exosomes secreted by glial cells can create a unique fertile environment suitable for the growth of cancer seeds (77–79). We guess that there are some inherent changes in CTCs that can limit the extracranial metastasis of gliomas. Changes in some epitope factors may occur when CTCs cross the blood-brain barrier. This is just our guess and needs further research to verify.

But this does not mean that it does not need to be taken seriously. There are still case reports of extracranial metastasis of glioma. Davis (80) for the first time fully reported cases of glioma concurrent with extracranial metastasis. Chang et al. (81) present a pathologically proven patient with scalp metastasis, which was metastasized from LGG occurring site to the surgical scar. And GBM metastases to the masticatory muscles and the scalp (82). There was even a case of a female patient with a known glioblastoma who was detected to harbor multiple metastases in the bones, lung, pleura, liver, mesentery, and the subcutaneous soft tissue (83). As the life span of patients with glioma increases after treatment, it is of great significance for detecting extracranial metastases. The research on CTC is hopeful to be an auxiliary diagnostic method for evaluating extracranial metastases in patients, and can be routinely screened for extracranial metastases in patients with glioma in clinical practice.

### Research on CTCs in Brain Metastases

In addition to gliomas, brain metastases account for a large proportion of intracranial malignant tumors. In non-small cell lung cancer (NSCLC), tumors metastasize most frequently to brain (40%) (84). 15–30% of patients with metastatic breast cancer will develop brain metastases during the course of the disease (85). Lung and breast cancer brain metastases are more commonly diagnosed than primary brain tumors. The formation of metastasis is the limiting factor of survival for most carcinoma patients. And Brain metastasis has devastating prognosis and accounts for significant morbidity and mortality in cancer patients (86). Brain metastasis are not only associated with an extremely poor prognosis but also with neurological impairments by often affecting both cognitive and sensory functions. Therefore, Brain metastasis have become a major limitation of life expectancy and quality of life in many patients.

Metastases originate from single or multiple tumor cells, circulating tumor cells (CTCs), which can initially be separated from the primary lesion, survive in the peripheral circulation and settle in distant target tissues (87). Studies have found that patients (NSCLC, colon cancer, SCLC, ovarian cancer, and renal cell carcinoma) with brain metastases have fewer CTCs than those with other metastases, and oligo- Metastatic patients have fewer peripheral blood CTC counts than multiple metastases (88). The number of CTCs in patients after brain metastasis resection is lower than that of patients without surgery or only radiotherapy and chemotherapy, and the number of CTC is significantly related to the survival of patients with brain metastases. These results indicate that the location and number of metastases have a strong influence on the number of CTCs. The detection of CTC has important clinical value for assessing the survival of patients with brain metastases, and even for identifying patients with brain metastases who may benefit from stronger treatments.

In addition, the recycling of CTCs from distant metastatic sites is also important. The recycling of CTCs seems to be more effective from other parts than the brain. This may be related to the existence of the blood-brain barrier, and some studies also speculate that a higher proportion of CTCs in patients with brain metastases may have undergone the epithelial-to-mesenchymal transition and cannot be detected (88). Carlotta et al. analyzed the copy number alteration (CNA) profiles of CTCs as well as autologous tumor tissue (89). Study has found that CTCs are similar to the corresponding primary breast tumors, but also found important pathway changes in brain metastasis, including notch (increased NOTCH3) and PI3K (increased PDPK1) pathways (90, 91). At the same time, the tumor cells metastasized to the brain have undergone strict clonal selection so that most of the CTCs in patients with brain metastasis exhibit a high degree of clonality. However, further studies need to be performed to validate these results. Research on CTC is not only helpful in evaluating the prognosis and treatment of patients with brain metastases, but also in understanding the complex mechanisms of brain metastasis at the molecular level.

## Conclusion

So far, there is limited data on CTC and brain tumors, and the application of CTC as a biomarker for glioma patients has only just begun. The identification of CTC has important clinical application value for early tumor diagnosis and prognosis. The integration of CTC and its molecular features in clinical practice may help to establish a diagnosis in a non-invasive manner and suggest complex tumor treatments without the need for high-risk neurosurgical intervention. The wide application prospect of CTC in glioma deserves further exploration, and the research on more sensitive and convenient detection methods is necessary.

## Search Strategy and Selection Criteria

Data for this Review were identified by searches of PubMed, and references from relevant articles using the search terms “Gliomas,” “Circulating tumor cells,” and “liquid biopsy.” Only articles published in English between 1990 and 2020 were included.

## Author Contributions

HZ collected data and wrote the paper. HZ, FQ, and YQ collect literature and information. QC and BL reviewed the paper. All authors read and approved the final manuscript.

## Conflict of Interest

The authors declare that the research was conducted in the absence of any commercial or financial relationships that could be construed as a potential conflict of interest.
